# Ferroelectric Domain Wall Memristor

**DOI:** 10.1002/adfm.202000109

**Published:** 2020-05-13

**Authors:** James P. V. McConville, Haidong Lu, Bo Wang, Yueze Tan, Charlotte Cochard, Michele Conroy, Kalani Moore, Alan Harvey, Ursel Bangert, Long‐Qing Chen, Alexei Gruverman, J. Marty Gregg

**Affiliations:** ^1^ Centre for Nanostructured Media School of Mathematics and Physics Queen's University Belfast Belfast BT7 1NN UK; ^2^ Physics and Astronomy University of Nebraska‐Lincoln Lincoln Nebraska 68588‐0299 USA; ^3^ Department of Materials Science and Engineering Pennsylvania State University 221 Steidle Building University Park PA 16802 USA; ^4^ Department of Physics School of Sciences and Bernal Institute University of Limerick Limerick V94 T9PX Ireland

**Keywords:** ferroelectric domain wall, memristor

## Abstract

A domain wall‐enabled memristor is created, in thin film lithium niobate capacitors, which shows up to twelve orders of magnitude variation in resistance. Such dramatic changes are caused by the injection of strongly inclined conducting ferroelectric domain walls, which provide conduits for current flow between electrodes. Varying the magnitude of the applied electric‐field pulse, used to induce switching, alters the extent to which polarization reversal occurs; this systematically changes the density of the injected conducting domain walls in the ferroelectric layer and hence the resistivity of the capacitor structure as a whole. Hundreds of distinct conductance states can be produced, with current maxima achieved around the coercive voltage, where domain wall density is greatest, and minima associated with the almost fully switched ferroelectric (few domain walls). Significantly, this “domain wall memristor” demonstrates a plasticity effect: when a succession of voltage pulses of constant magnitude is applied, the resistance changes. Resistance plasticity opens the way for the domain wall memristor to be considered for artificial synapse applications in neuromorphic circuits.

## Graphical abstract

By changing the density of conducting ferroelectric domain walls that straddle the interelectrode gap, it is shown that a large number of different direct current resistance states can be created, in parallel‐plate thin film lithium niobate capacitors. Surprisingly, such microstructural manipulation can result in colossal changes in device resistance (over twelve orders of magnitude).

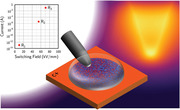

## 1 Introduction

Domain walls in ferroelectrics and multiferroics have functional properties that are often distinct from those of the domains that they surround.^[^ ^1^, ^2^, ^3^, ^4^, ^5^, ^6^ ^]^ Enhanced electrical conductivity, in comparison to bulk, has been observed in many systems^[^ ^3^, ^4^, ^5^, ^6^, ^7^, ^8^, ^9^, ^10^, ^11^, ^12^, ^13^, ^14^, ^15^, ^16^ ^]^ and this has inspired researchers to imagine how switching‐controlled injection, movement and annihilation of conducting walls (to make or break electrical connections) might facilitate new kinds of dynamic nanocircuitry.^[^ ^17^, ^18^, ^19^, ^20^, ^21^ ^]^ Early‐stage devices have recently emerged: for example, studies on bismuth ferrite have revealed domain‐wall enabled memory, where binary states have been switched by AFM contact to an electrode^[^ ^22^, ^23^ ^]^; other research,^[^ ^24^ ^]^ on lead zirconate titanate, has demonstrated transient conducting domain wall conduits, providing a volatile (non‐remnant) resistance change in a transistor‐like device. In both of these studies, only two or three resistance states have been accessed, based on a single pair of conducting domain walls straddling an inter‐electrode gap. Conceptually, however, the number of distinct resistance states in such devices could be almost without limit: as long as different populations of conducting domain walls are injected in a controllable and repeatable manner, then there should be no problem in developing domain‐wall‐enabled multi‐resistance devices, or domain wall memristors (electrical circuit components that behave like a non‐linear resistor with memory, described by a relation between integrated potential difference and flux linkage^[^ ^25^, ^26^ ^]^). A proof‐of‐concept demonstration has already been performed in BiFeO_3_, by Siedel et al.,^[^ ^4^ ^]^ where different numbers of conducting domain walls were manually written in pairs between electrodes, using an atomic force microscope (AFM). While illustrating the viability of the concept, such experiments are clearly a long way from practical devices.

In this letter, we report a major step forward in harnessing conducting domain walls as the active components in new forms of memristors. We demonstrate devices, based on simple parallel‐plate capacitor structures, in which thin film ion‐sliced single crystal congruent lithium niobate acts as the dielectric (ferroelectric) layer. Partial reversal of the polarization in the ferroelectric causes the steady‐state resistance of the capacitor to change by up to twelve orders of magnitude. This change is caused by the injection of strongly inclined conducting 180^°^ domain walls, the density of which can be controlled by the extent to which ferroelectric switching has progressed. Since this is, itself, a function of the electric field (the polarization‐field hysteresis loop is a defining characteristic of a ferroelectric), a large range of resistance states can be accessed by applying voltage pulses of different magnitudes: indeed, we explicitly demonstrate that at least 100 stable conductance states can be induced within a two order of magnitude window in conductance, using writing pulses of fixed length and variable amplitude. Conductance of the device can also be progressively changed by applying multiple successive voltage pulses of the same magnitude: a phenomenon known as “plasticity” which can, in principle, be utilized in artificial synapse development. While the details of the conduction mechanisms involved are radically different, the domain wall memristors that we demonstrate herein are reminiscent of those based on asymmetric ferroelectric tunnel junctions:^[^ ^27^, ^28^ ^]^ in both cases different conductivity states are made by controllably changing the relative volume fraction of differently oriented domains.

## 2 Lithium Niobate

Lithium niobate (LiNbO_3_) is an archetypal trigonal uniaxial ferroelectric, used extensively for applications in non‐linear optics and in surface acoustic wave devices. It has a high Curie temperature (between 1141 and 1210 °C) and a commensurately large room temperature spontaneous polarisation (≈70 µC cm^−2^) that varies significantly with stoichiometry.^[^ ^29^, ^30^ ^]^ Under ambient conditions, the bulk single crystal is considered to be a perfect insulator^[^ ^31^, ^32^ ^]^ and this makes the contrast in transport behavior between domains and charged conducting domain walls particularly stark. LiNbO_3_ is usually non‐stoichiometric, as single crystal growth from the congruent melt leads to lithium deficiency and subtle associated defect chemistry.^[^ ^33^, ^34^ ^]^ Even in bulk, the coercive field needed for ferroelectric switching can be as high as 220 kV cm^−1^ and imprint, due to defect‐related internal fields, is common. In thin films, coercive fields increase and imprint effects are often exaggerated:^[^ ^35^, ^36^, ^37^, ^38^ ^]^ Volk et al.^[^ ^39^, ^40^ ^]^ found coercive fields in 300 nm thick ion‐sliced layers to be ≈670kV cm^−1^ (around three times that of bulk), while Jiang et al.^[^ ^41^ ^]^ found such strong imprint that only one remnant polarization state could be stabilized. For the research described herein, commercially obtained thin film material was used. The as‐received structure consisted of a 500 nm thick single crystal lithium niobate layer (ion‐sliced from a congruent boule) bonded to a chromium–gold–chromium thin film electrode (150 nm thick) above a silica adhesion layer (2000 nm), on top of a bulk single crystal lithium niobate wafer. The ion‐sliced layer was such that the polarization was oriented perpendicular to the film surface (z‐cut).

Enhanced conductivity along 180° domain walls in lithium niobate had been suspected for some time (by Mizuuchi et al.^[^ ^42^ ^]^ for example), but is now a well‐established phenomenon: pioneering work by Lukas Eng and coworkers initially observed that domain wall conduction could only be induced by photoexcitation,^[^ ^12^ ^]^ but they later found that, even in the absence of light, walls that were inclined with respect to the polarization axis would conduct.^[^ ^13^ ^]^ Inclined walls are necessarily charged, as they create discontinuities in polarization, seen in other ferroelectric systems to be critical for generating or suppressing domain wall currents.^[^ ^7^, ^8^, ^9^, ^14^, ^15^, ^43^ ^]^ In lithium niobate, head‐to‐head polar discontinuities cause enhanced domain wall conduction, but the inclination angles seen vary widely: in some studies, complete “inversion” domain walls can be created, with effectively 90° inclination angles between the domain wall and the polarization vector. However, “inversion” layers require exotic processing routes, such as extreme heat treatments (typically to ≈1000 °C^[^ ^44^ ^]^), partial melting,^[^ ^45^ ^]^ or ion diffusion, sometimes in the presence of molten salts.^[^ ^46^ ^]^ While worth noting their existence, it is clear that the conditions needed to make them are incompatible with standard electronic device operation. When device compatible electric‐field switching processes are used, the maximum inclination angles reported to date have been modest and of the order of 1–3°; nevertheless, this still allows significant domain wall conduction to occur.^[^ ^13^, ^20^ ^]^

## 3 Results and Discussion

### 3.1 Switching Single Domains and Observing Conducting Domain Walls

**Figure 1:**
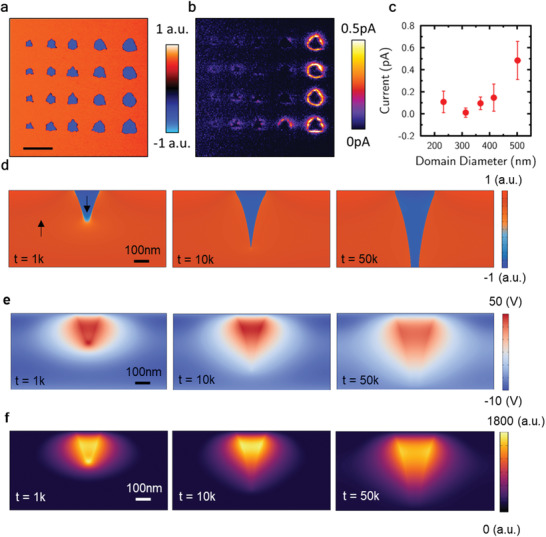
a) Vertical piezoresponse force microscopy map of a 5 × 5 µm^2^ region of the 500 nm thick ion‐sliced lithium niobate single crystal, showing the combined vertical amplitude and phase response of the domain structure written using a stationary biased atomic force microscopy tip. b) Conductance map of the same region with −2.3 V DC bias applied to the bottom electrode. c) A plot of current against domain diameter, as measured at the top surface of the LiNbO_3_ thin film. Points represent mean currents obtained from the 100 datapoints at each domain size which showed the largest current values (error bars mark two standard deviations). d–f) Phase‐field simulation of d) domain evolution during tip‐induced switching in a 500 nm LiNbO_3_ film and corresponding e) electrostatic potential and f) electron density, estimated by using a degenerate electron gas approximation.

As a general observation, 180° domain walls, written into the as‐received monodomain LiNbO_3_ films used in our study, showed dramatically enhanced conduction compared to the domains themselves (see Figure S1, Supporting Information). This is commensurate with reports in established literature. Surprisingly, though, when nanodomains were written, using a stationary AFM tip, the measured domain wall currents (**Figure** **1**a–c) were found to be strongly dependent on the domain diameter, as observed on the top surface (controlled by tip‐poling, using different voltage pulse lengths). An apparent threshold behavior was evident, in which domain wall conduction increased dramatically for top surface domain diameters above ≈400 nm. 180° switching in lithium niobate proceeds through the nucleation and growth of needle domains, with necessarily inclined domain walls.^[^ ^12^, ^13^ ^]^ Usually, growth is seen to be a process with two distinct stages: initially, forward propagation of the needle domain occurs, until it reaches the opposite electrode; subsequently, switching progresses through sideways domain wall motion. Under these circumstances a sudden increase in measured conduction should be expected at the point at which the needle domain first contacts the opposite electrode, completing the low resistance domain wall current pathway. Given the thickness of the lithium niobate film is 500 nm and wall currents were found to suddenly increase at a threshold top surface domain diameter of ≈400 nm, an extremely high inclination angle for the needle domain of over 20° is implied by simple geometry (assuming a conical needle domain). This result is very surprising, given that previously considered “high” inclination angles had a mean value of ≈1°^[^ ^13^, ^20^ ^]^ but it is also exciting, as it suggests that the conductivity of the domain walls in these ion‐sliced systems can be particularly high.

### 3.2 Phase‐Field Simulation of Domain Switching Kinetics and Domain Wall Tilt Angles

To gain further insight into the existence of such unusual domain wall inclination angles, we performed phase‐field simulations in a 2D cross‐sectional model system of the LiNbO_3_ film. The simulation starts with a single domain state with upward polarization. A local bias is then applied from a tip at the surface, and the resultant domain switching process is recorded and visualized (in Figure 1d). We can see the typical switching stages, including domain nucleation, forward and sideways growth. Note that the domain wall tilt during the whole process is 10–15°. At each snapshot of domain evolution, we also calculate the electrostatic potential distribution and estimate local free electron density from that using the degenerate electron gas approximation.^[^ ^47^ ^]^ The enhanced local electrical potential and subsequent accumulation of free carriers near the tilted domain walls are plotted in Figure 1e,f, respectively. A clear correlation between the local free carrier concentration and the tilted domain wall angle can be seen. Note that, although the free carrier density is higher at the early stage when the domain begins to nucleate, the domain conduction occurs only when the switched domain penetrates the whole film thickness, corresponding to a tilt angle of ≈12°. While this is not as dramatic as that estimated from the simple geometric arguments discussed above, it still suggests that domain walls with extreme inclination angles have been introduced into the system and that these domain walls should be particularly conductive. What is perhaps unusual is the fact that the large inclination angles, modelled to exist during the switching process, are maintained in the steady‐state once the switching field has been removed.

### 3.3 Parallel‐Plate Switching and Cross‐Sectional Domain Wall Imaging

**Figure 2:**
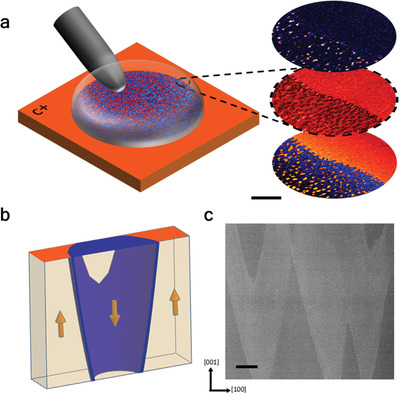
a) Schematic showing a tungsten carbide needle electrically contacting microscopic Ga–In–Sn eutectic alloy electrodes to allow switching and conductance measurements at the mesoscale. The three images to the right are (top‐to‐bottom) typical current, piezo‐response amplitude and phase across the boundary between a partially switched and completely unswitched region. The fine‐scale microstructure and localized conducting domain walls are evident. The scale bar for the maps is 1 µm. b) 3D schematic showing typical domain structure in a lamella (arrows represent polarization directions) used for transmission electron microscopy investigations. c) Scanning transmission electron microscopy high angular annular dark field image (STEM‐HAADF) of a lamella cut from a partially switched region. The high density and angle of the domain walls can clearly be seen. The variations in contrast are associated with variations in the microstructure that occur through the thickness of the lamella: dark areas are associated with a uniform domain state (either “up” or “down”) throughout the entire lamellar thickness (cream regions in the schematic shown in (b), while lighter areas are associated with both “up” and “down” domains and the domain wall being sampled by the electron beam in the through‐thickness direction (in the schematic shown in b, these would be areas in which the blue domain wall is sampled in the through‐thickness direction). Scale bar 50 nm.

The properties of mesoscale parallel‐plate capacitor structures were then examined, using a liquid indium–gallium–tin alloy droplet as a top electrode. In **Figure** **2**a, a schematic of the experiment geometry is combined with piezo‐response force microscopy and conducting AFM data for a partially switched configuration (imaged after the electrode had been removed). Of particular note is the fine scale of the microstructure (of the order of ≈50 domains per square micron), for which the associated domain wall density is necessarily large. Cross‐sectional scanning transmission electron microscopy (STEM) on these samples confirms directly that the domain wall inclination angles are significant (Figure 2c and the schematic representation in Figure 2b), measured to be between 10 and 15°, consistent with our phase field modelling. We note that similar microstructures were created when more conventional gold and silver thin film top electrodes were used.

**Figure 3:**
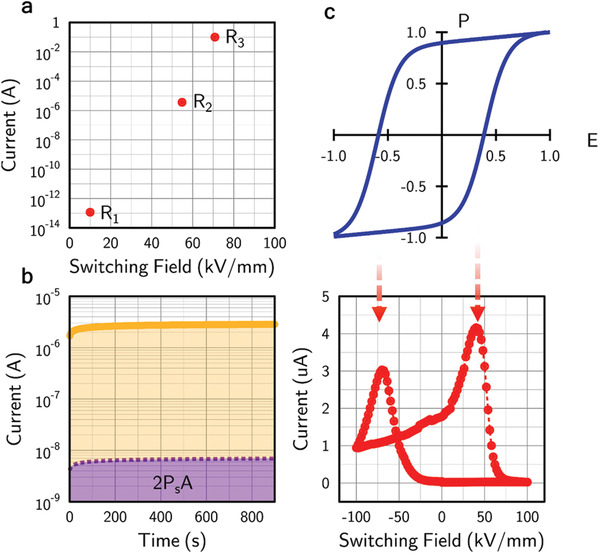
a) Three different conduction states induced during initial switching studies, spanning twelve orders of magnitude in resistance. The current measured for the R_3_ state hit the compliance limit of the source‐measure unit, implying that even greater resistance changes during switching are likely to occur. b) Steady‐state current (measured at 10 kV mm^−1^) in a high conductance state (yellow/orange) compared with the equivalent switching current based on the maximum possible switching charge discharged over the 900 s measurement period (purple). Clearly, the dc currents observed cannot be accounted for by domain wall movement. c) A schematic hysteresis loop compared with the conductance measured as a function of switching field. Currents were measured at 5 V (equivalent field of 10 kV mm^−1^) applied for 1000 ms and the switching field was applied stroboscopically (in between each dc current measurement) in 10 ms pulses. Arrows indicate the relation between the current peaks (states of highest conductance) and the coercive field in the P–E loop. Note the slight imprint which causes off‐centring of the P–E response and an asymmetry in the switching fields needed to generate the most highly conducting states.

The combination of high domain wall density and strongly inclined domain wall geometry is likely to be responsible for the dramatic changes in resistance of these parallel‐plate capacitors seen during partial switching experiments: as shown in **Figure** **3**a, initial measurements (using large top electrode indium–gallium–tin droplets) found resistance during switching to decrease by over twelve orders of magnitude from that of the as‐received monodomain state. Further switching (into even more conductive states) was impossible in these early‐stage investigations, as the current compliance limit on our power source had been reached. The measurement problem was overcome by using smaller top electrodes (1.5 × 10^4^ µm^2^), allowing switching pulses of up to 100 kV mm^−1^, which were sufficient to traverse almost the entire polarization‐field hysteresis loop for the lithium niobate films (Figure 3c). Between each switching pulse, the steady‐state dc current (under a 10 kV mm^−1^ field) was monitored. Its form is as might be expected (Figure 3c): low measured dc currents were associated with almost fully‐poled states, with few domain walls, while high currents were found around the apparent coercive field, where the largest domain wall densities should be expected. To explore further, microstructures were explicitly examined after the LiNbO_3_ had been exposed to a range of different voltage pulse magnitudes. Particular attention was focused on those developed on approaching the apparent coercive field (Figure S2, Supporting Information). Images confirm that microstructures which support the largest steady‐state dc currents are highly mixed (with around equal proportions of the two domain orientations present). Switching fields, above and below the coercive field (Figures S2 and S3, Supporting Information), still produce mixed microstructures (commensurate with the non‐abrupt switching in the schematic polarization‐field hysteresis loop shown in Figure 3c), but it is clear that the relative proportions of the two domain states change as the coercive field is approached and exceeded, as should be expected.

To show that the measured conductances were due to genuine charge transport (as opposed to switching currents resulting from domain wall movement), the dc current as a function of time for a partially switched state is shown in Figure 3b and is compared to the maximum switching current that could have been generated over the same time from complete ferroelectric polarization reversal. As can be seen, the dc currents observed are several orders of magnitude greater than the maximum level that could have arisen from switching currents and so are clearly due to genuine domain wall transport. Nevertheless, the transient injection currents must be important for ensuring that the strongly inclined domain walls, characteristic of the microstructures observed, remain stable:^[^ ^48^ ^]^ given the significant wall inclination angles and the large domain wall densities developed during switching, charge injection of the order of ≈50 nC would be needed just to stabilize the walls. This will be manifest as part of the switching current, however, and so is not relevant to the large dc currents described above.

### 3.4 Memristor Behaviour

**Figure 4:**
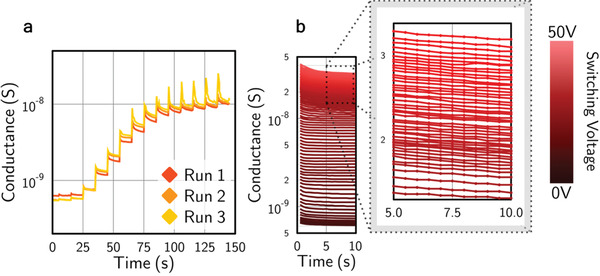
a) Conductance variations with time when switching field pulses (1000 ms duration) of progressively increasing magnitude (in 4 V steps) are applied every 10 s: in this case, at least ten different stable remnant states have been demonstrated within an order of magnitude change in conductance b) Finer steps of increasing pulsed switching voltage, varied between 0 and 50 V (in 500 mV steps), generated 100 different conductance states within a two orders of magnitude window in conductance. For each state, the time dependence of the conductance is presented, monitored for 10 s after the completion of the switching pulse used to create it. Inset: higher resolution plot of the states created by switching pulses at the upper end of the voltages used.

An exploration of the density of distinct remnant conductance states that might be generated in this domain‐wall enabled memristive device was undertaken (illustrated in **Figure** **4**). Switching pulses of progressively increasing magnitude were applied and the resultant conductance levels produced were monitored as a function of time. Even with rather crude step‐increases in switching fields (Figure 4a), we were able to demonstrate that ten distinct conductance levels could be written within an order of magnitude window in device conductance. This density of written states could be dramatically increased by using finer differences in the magnitudes of applied switching pulses: Figure 4b shows that 100 distinct memristive states could be written within a conductance range of less than two orders of magnitude in size. In principle, extrapolating across the entire range of conductances seen in these LiNbO_3_ films (Figure 3a), each domain wall memristor could therefore be set to one of over 500 distinct states.

An obvious feature, associated with creating each distinct conductance state, is a short‐lived transient relaxation, which appears to be exponential in nature. The magnitude of this relaxation varies with the magnitude of the increase in voltage between successive writing pulses (the effect is much more noticeable in Figure 4a than in Figure 4b, for example). We are uncertain as to the origin of this effect, but suspect it to be a capacitive transient. It does not seem to affect the conduction state of the partially switched microstructures over significant timescales. Indeed, we note that the conductance states appear to be remarkably stable: we notice little change over periods of days to weeks. This contrasts strongly with observations made previously by Lu et al.^[^ ^49^ ^]^ on similar domain walls, where a progressive relaxation of the wall inclination angle within a region close to the LiNbO_3_ surface completely shut‐down all dc conduction. We suspect that, the “read” voltages that we use to monitor conductivity in our devices are sufficient to reverse any relaxations in domain wall inclination angles, if they do occur. This would be consistent with the “wake‐up” observations also made by Lu et al.

**Figure 5:**
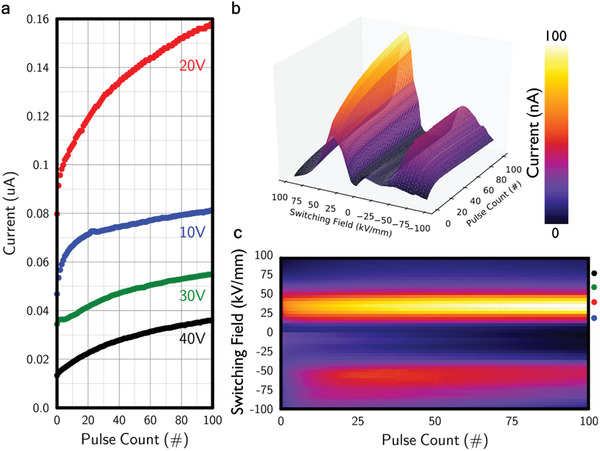
a) A plot of current measured under a 5 V steady‐state potential difference (10 kV mm^−1^ field) against the number of 10 ms pulses applied, with the voltages of these pulses as labeled. The domain state was reset before each set of measurements made with a given pulse voltage magnitude. b) A 3D plot showing steady‐state currents as a function of the cumulative number of pulses applied, for different pulse voltage magnitudes. The double peak features that emerge mirror those in Figure 3c, but here the plasticity effects are clearly evident. c) Shows (b) from a bird's‐eye view. Colored dots indicate the switching pulse fields associated with data in (a).

An interesting functional response, seen previously in ferroelectric tunnel junction memristors,^[^ ^50^, ^51^ ^]^ is that of “plasticity” of conductance, where the resistance of the device can be progressively altered by exposure to sequential switching voltage pulses of constant magnitude. **Figure** **5** shows how our domain wall memristors respond to up to 100 identical pulses, as a function of switching field. A plasticity effect is obvious: the low field dc conductance progressively increases as the number of constant voltage switching pulses increases, for all levels of pulse voltage. The plasticity effect is most pronounced around the coercive field.

## 4 Conclusions

In conclusion, we have demonstrated a ferroelectric memristor in which different remnant conducting states result from different densities of conducting domain walls in the microstructure. The number of distinct addressable states is significant (of the order of hundreds in each device). Plasticity in the conductance, as a function of the number of pulses applied to these domain wall memristors, could be relevant in applications such as artificial synapses, so that they could influence aspects of future neuromorphic computing. There are, of course, potential limitations: for example, the domain microstructures are hundreds of nanometers in length scale and this would currently prevent significant electrode miniaturization. However, we expect that much thinner LiNbO_3_ films should support commensurate reductions in the scales of the domains induced during switching. Exploration of this possibility will be one of the subjects for further future work.

## Conflict of Interest

The authors declare no conflict of interest.

## Supporting information

Supporting Information
