# Supplementary material for: Ferroelectric Domain Wall Memristor
Source: Adv Funct Mater. 2020 May 13;30(28):2000109. doi: 10.1002/adfm.202000109 (PMC7357591; doi:10.1002/adfm.202000109)
Supplement: Supplementary file 1 — Supporting Information [file ADFM-30-2000109-s001.pdf]

Copyright WILEY-VCH Verlag GmbH & Co. KGaA, 69469 Weinheim, Germany, 2020.

ADVANCED FUNCTIONAL MATERIALS

Supporting Information

for *Adv. Funct. Mater.,* DOI: 10.1002/adfm.202000109

Ferroelectric Domain Wall Memristor

*James P. V. McConville, Haidong Lu, Bo Wang, Yueze Tan, Charlotte Cochard, Michele Conroy, Kalani Moore, Alan Harvey, Ursel Bangert, Long-Qing Chen, Alexei Gruverman, and J. Marty Gregg**

Supplementary Information

Figure S1 | AFM images of a domain switched using a tungsten needle (**a**) Topographic map of a 5x5um region of the 500nm thick ion-sliced lithium niobate single crystal, measured with atomic force microscopy. (**b**) Vertical piezo-response map from the same area, showing the combined amplitude and phase response of the domain structure with c+ and c- labelling polarisation out-of and into the plane respectively. (**c**) Conductance map measuring current locally with -4V DC bias applied to the base of the sample through the gold and chromium electrode (contacted with conducting silver paint). The poling procedure is described in the method section of the main paper. The scale bar in (**a**) is 1um and all images use the same scale.

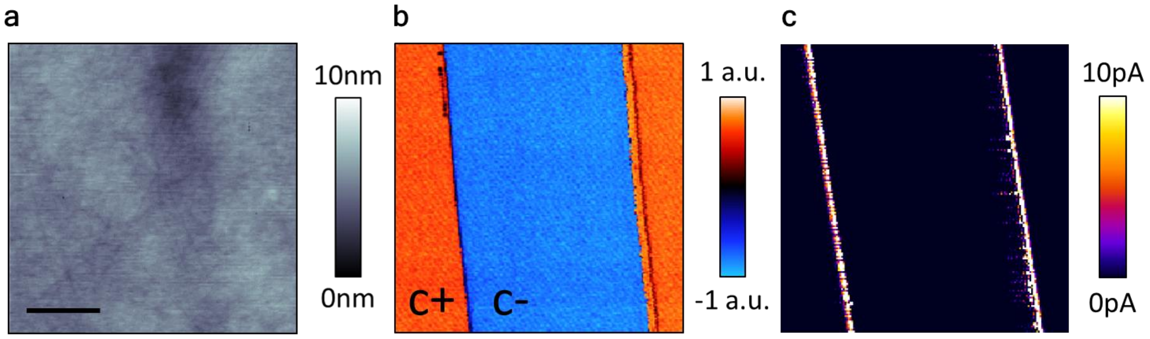

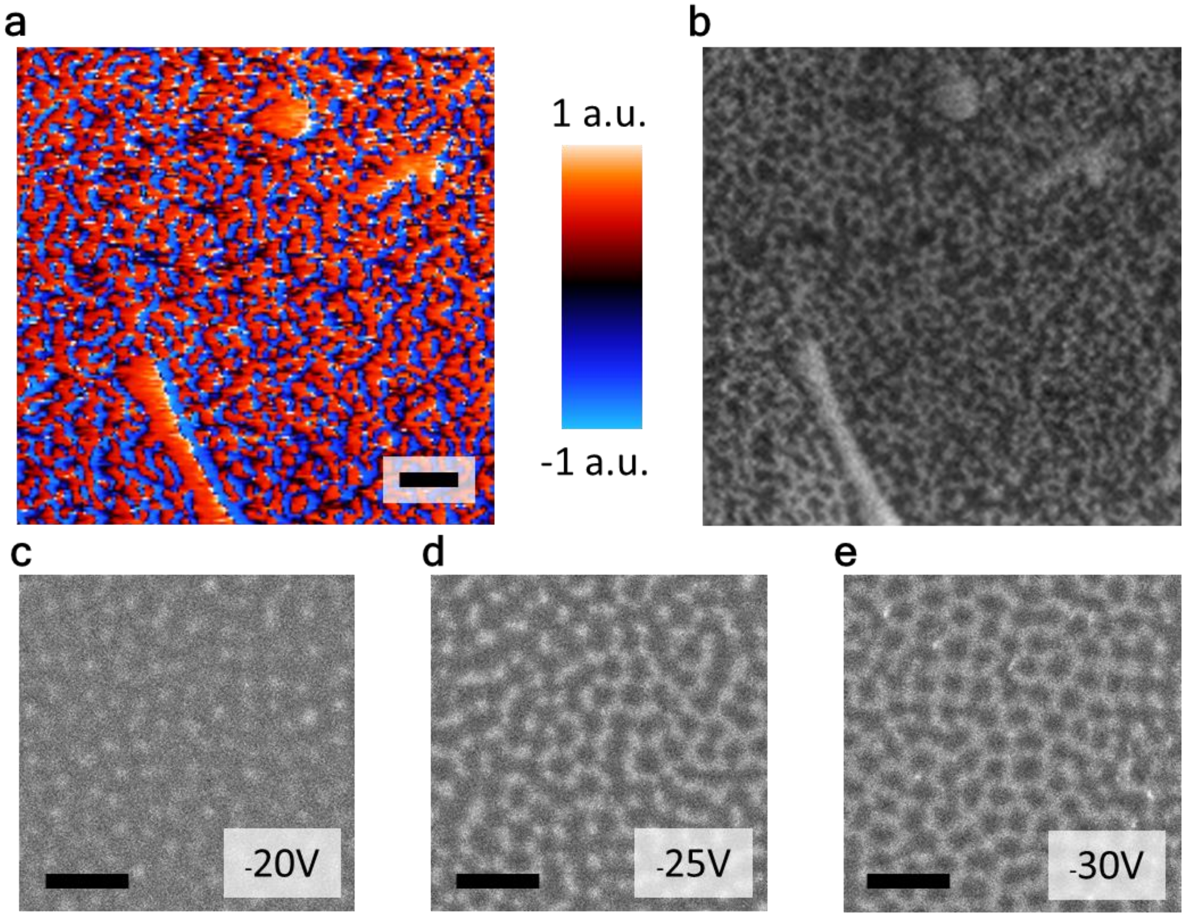

Figure S2 | AFM images of the domain microstructure switched using a eutectic alloy top electrode (**a**) Piezo-response phase map obtained from a partially switched region of the lithium niobate thin film (after removing the alloy top electrode using a mechanical polish). (**b**) Scanning Scanning electron microscope (SEM) image of the same region showing that the same domain contrast can be obtained using an in-beam secondary electron detector. (**c-e**) Representative SEM micrographs showing broadly how the domain structure evolves as the switching voltage pulse magnitude increases. Domain states are distinctly mixed, with the relative populations of the two domain orientations obviously changing as a function of applied voltage, but not in a discontinuous manner. Such observations are consistent with the tilted P-E hysteresis loop shown schematically in figure 3(**c**) in the main paper. All scale bars indicate 500nm.

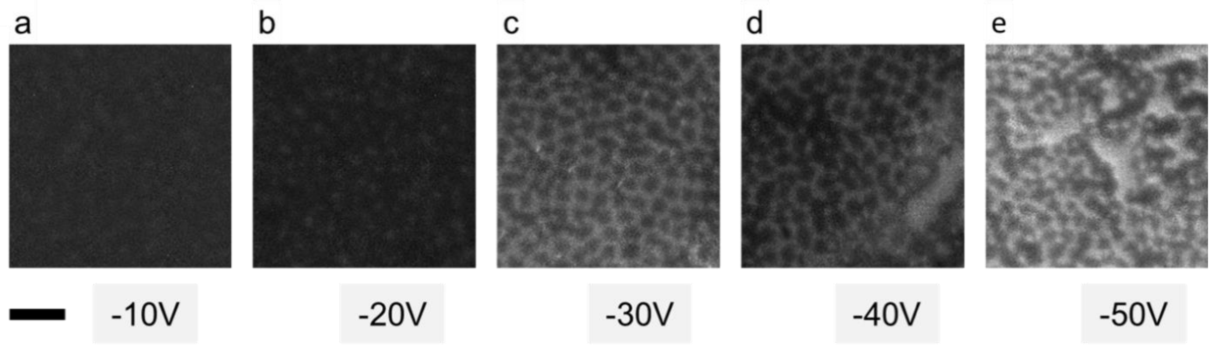

Figure S3 | SEM images of the domain microstructures developed under different applied voltages (**a-e**) Representative SEM micrographs show how the domain structure evolves with the indicated increasing negative switching voltages applied stroboscopically, as in figure 3(**c**) of the main paper. It is clear that the relative fraction of domains with light contrast increases as the magnitude of the switching voltage pulse increases. Scale bar indicates 500nm and all images are the same scale. The contrast is not normalised.

Details on Methods:

Sample Preparation: All measurements were performed on sections of two wafers of ion-sliced congruent lithium niobate obtained from NanoLN. The 3 inch diameter wafers each consist of a 500nm thick single crystal of congruent undoped z-cut lithium niobate, bonded to a 150nm thick chromium-gold-chromium electrode, a 2μm silica underlayer and finally a 0.5mm thick substrate of z-cut lithium niobate. Conductive silver paint was used to contact the bottom electrode for all electrical measurements. Various contacts to the top surface were used as described below.

Domain switching: Domains were switched using various top contacts. The domains in Figure 1 were switched with a DC bias of 50V applied to whole platinum atomic force microscopy probes for varying durations, described below (see the scanning probe section of the Methods).The domains in Figure S1 were switched with a DC bias of 50V applied to a tungsten carbide micromanipulator needle with tip radius of 10μm, moved across the crystal at a constant rate of 0.1mms^{-1}.

Scanning probe microscopy imaging: PFM and c-AFM imaging in Figure 1 were performed using an MFP-3D AFM system (Asylum Research) using PPP-EFM tips. The PFM images were obtained at AC modulation bias of 0.6 V in amplitude, at 350 kHz. The c-

AFM images were obtained with DC bias of -2.3 V applied to the bottom electrode. Domain patterns in Figure 1 were created by application of +50V to the tip, with different pulse durations (1ms, 10ms, 100ms, 1s and 10s).

PFM and topography maps in Figure 2 and Figure S1 were captured simultaneously with a Veeco Dimension 3100 AFM system with a Nanoscope IIIa controller. An EG&G 7256 lock-in amplifier is used to apply an AC bias of 2VRMS with a frequency of 20 kHz to the base of the sample via a silver paste bottom electrode for PFM measurements. The c-AFM characterisation in Figure S1 was obtained on the same system using an additional Bruker Tunnelling AFM (TUNA) module. A DC bias of -4V was applied to the bottom electrode and the tip grounded. Commercially obtained platinum/iridium coated silicon probes (Nanosensors model PPP-EFM) were used with a force constant of 2.8Nm^{-1} for all data in Figure 2 and S1.

Electron Microscopy: Cross-sectional transmission electron microscopy (TEM) specimens were prepared using a dual-beam focused ion beam (FIB) integrated scanning electron microscope (Thermo-Fisher Scientific FEI Helios 660). The specimen was mounted onto an Ominprobe® copper-based lift-out grid. The scanning TEM (STEM) analysis was performed using a Thermo-Fisher Scientific double tilt TEM holder in the Thermo-Fisher Scientific FEI double aberration-corrected monochromated Titan Themis Z at University of Limerick. The microscope was operated at 300 kV.

Scanning electron microscope (SEM) micrographs were obtained using dual-beam focused ion beam (FIB) integrated scanning electron microscope (Tescan LYRA). The bottom electrode was grounded and low beam currents were used to minimise charging. Domain contrast was noted to be stronger using the in-beam secondary electron detector.

Conductance Measurements of Capacitor Structures: For all remaining figures, samples were switched according to the schematic in Figure 2. A tungsten carbide needle was used to apply and contact small eutectic alloy electrodes (100μm x 100μm typically). Current measurements were performed using a Keithley model 237 Source-Measure Unit.

Phase Field Modelling: The phase-field simulations of 500 nm $LiNbO_3$ are performed in a quasi-2D system with a discretized grid of $1024\Delta\mathrm{x} \times 2\Delta\mathrm{y} \times 512\Delta\mathrm{z}$ ($\Delta\mathrm{x} = \Delta\mathrm{y} = \Delta\mathrm{z} = 1$ nm). The AFM tip bias at the film surface is approximated by a Lorentz distribution with $(r) = \frac{\lambda^2\phi_0}{\lambda^2+r^2}$, where $\lambda = 200$ nm, $\phi_0 = 200$ V, and r is the distance from the contact center. The

TDGL equation for polarization evolution, the Poisson equation for electrostatics, and the mechanical equilibrium equation are solved self-consistently in the framework developed in our previous works [1,2]. The stress-free and clamped mechanical boundary conditions are assumed for film surface and bottom, respectively, whereas short-circuit electrical boundary conditions are used for both interfaces. The materials parameters, including Landau coefficients, elastic properties, and background dielectric constants of $LiNbO_3$, are adopted from previous publications [3,4,5]. The carrier density (free electrons) mapping at equilibrium near the tilted domain walls are calculated with the degenerate electron gas approximation [3].

[1] Y. L. Li, S. Y. Hu, Z. K. Liu, L. Q. Chen, Acta Mater., **50**, 395-411 (2002).
[2] L. Q. Chen, J. Am. Ceram. Soc., **91**, 1551-2916 (2008).
[3] E. A. Eliseev, A. N. Morozovska, G. S. Svechnikov, V. Gopalan, and V. Y. Shur Phys. Rev. B **83**, 235313 (2011)
[4] D. A. Scrymgeour, V. Gopalan, A. Itagi, A. Saxena, P. J. Swart, Phys. Rev. B **71**, 184110 (2005).
[5] A. N. Morozovska, E. A. Eliseev, Y. Li, S. V. Svechnikov, P. Maksymovych, V. Y. Shur, V. Gopalan, L.Q. Chen, S.V. Kalinin, Phys. Rev. B **80**, 214110 (2009).
